# A numerical study of the effect of thrombus breakdown on predicted thrombus formation and growth

**DOI:** 10.1007/s10237-023-01757-8

**Published:** 2023-08-11

**Authors:** Kaihong Wang, Chlöe H. Armour, Richard G. J. Gibbs, Xiao Yun Xu

**Affiliations:** 1 Department of Chemical Engineering, Imperial College London, London, UK; 2 Regional Vascular Unit, St Mary’s Hospital, Imperial College Healthcare National Health Service Trust, Imperial College London, London, UK

**Keywords:** Thrombosis modeling, Computational fluid dynamics, Hemodynamics, Backward-facing step, Thrombus breakdown, Shear stress

## Abstract

Thrombosis is a complex biological process which involves many biochemical reactions and is influenced by blood flow. Various computational models have been developed to simulate natural thrombosis in diseases such as aortic dissection (AD), and device-induced thrombosis in blood-contacting biomedical devices. While most hemodynamics-based models consider the role of low shear stress in the initiation and growth of thrombus, they often ignore the effect of thrombus breakdown induced by elevated shear stress. In this study, a new shear stress-induced thrombus breakdown function is proposed and implemented in our previously published thrombosis model. The performance of the refined model is assessed by quantitative comparison with experimental data on thrombus formation in a backward-facing step geometry, and qualitative comparison with in vivo data obtained from an AD patient. Our results show that incorporating thrombus breakdown improves accuracy in predicted thrombus volume and captures the same pattern of thrombus evolution as measured experimentally and in vivo. In the backward-facing step geometry, thrombus breakdown impedes growth over the step and downstream, allowing a stable thrombus to be reached more quickly. Moreover, the predicted thrombus volume, height and length are in better agreement with the experimental measurements compared to the original model which does not consider thrombus breakdown. In the patient-specific AD, the refined model outperforms the original model in predicting the extent and location of thrombosis. In conclusion, the effect of thrombus breakdown is not negligible and should be included in computational models of thrombosis.

## Introduction

Thrombosis is a complex biological process involving many biochemical reactions that occur in injured vessels throughout the human body. The degree of thrombosis can impact patient prognosis and treatment outcomes either positively or negatively depending on where it occurs (Anand et al. 2005; Hansen et al. 2015; Cito et al. 2013). Complete false lumen (FL) thrombosis in aortic dissection (AD) indicates favorable outcomes in aortic remodeling (Bernard et al. 2001), whereas thrombus formation in a wrong place can lead to serious complications, such as obstructive thrombosis in small vessels (De Silva and Faraci 2016; Roudaut et al. 2007; Strueber et al. 2014).

Thrombus formation is a consequence of abnormal hemostasis in damaged vessels, and interactions among platelets, the vessel wall and chemical factors play important roles in thrombosis (Schenone et al. 2004; Fernández-Ortiz et al. 1994). In damaged regions, subendothelial cells are exposed to blood flow, and long-time exposure can trigger a series of biochemical reactions among tissue factors (TF) and blood cells, such as von Willebrand factor and fibronectin, to activate platelets and promote platelet adhesion. The coagulation cascade is also triggered at the same time, allowing fibrin formation which facilitates clotting process and thrombus growth (Mosesson 2005; Brass 2003). Furthermore, fibrinolysis occurs and works to regulate thrombus growth rate and remodel thrombus by a series of enzymatic reactions (Austin 2017).

Abnormal hemodynamics have a strong influence on thrombosis in damaged regions. It has been recognized that elevated wall shear stress (WSS) can activate platelets, whereas flow stasis favors platelet adhesion and accumulation. Initial thrombus deposition starts when activated platelets travel to regions of flow stasis and then adhere to the vessel wall. On the other hand, clot lysis can occur when formed thrombus is exposed to high-shear-stress flow over a prolonged time period (Riha et al. 1999; Dimitrov et al. 2020; Tippe and Müller-Mohnssen 1993).

The desire to understand such a complex process in cardiovascular diseases has driven the development of computational models to predict thrombosis in recent years, and a physiologically realistic and accurate thrombosis model is essential to aid clinicians in decision-making process. To date, computational models have successfully simulated thrombus formation based on either kinetics or hemodynamics methods. Kinetics-based models describe a series of key biochemical reactions that occur during thrombosis. A TF-initiated model was proposed to simulate the coagulation system by incorporating both blood coagulants and anticoagulants (Hockin et al. 2002). This was further modified by including additional anticoagulants: protein C, AT-III, and TF pathway inhibitor, to inhibit coagulation (Fogelson and Tania 2005). Inhibition of coagulation slowed down the clotting process and worked to modulate the thrombus growth rate. Another way to regulate thrombus growth is clot lysis. Anand et al. (2005) proposed a clot lysis model where the onset of clot lysis was modulated by the concentrations of fibrin (Ia), protein C and tPA, among others (Anand et al. 2005, 2003). Due to the large number of reactants included, which led to a highly nonlinear system of coupled equations, this model was computationally expensive, making it difficult to be applied to complex geometries and pulsatile flows.

Compared with kinetics-based thrombosis model, hemodynamics-based models are less computationally demanding. In various models reported in the literature, the coagulation process has been simplified by modeling a limited number of representative coagulants, with platelet activation, aggregation and thrombus development being regulated by local hemodynamic conditions (Anand et al. 2003; Menichini and Xu 2016; Taylor et al. 2016; Goodman et al. 2005; Fogelson and Tania 2005; Fogelson and Guy 2008; Longest and Kleinstreuer 2003; Armour et al. 2022). In Menichini and Xu’s model (2016), platelets were modeled in three states (resting, active, and bound), and platelet activation was controlled by the exposure time to thrombin and already activated platelets. Thrombin was not specifically modeled; instead, thrombin concentration was assumed to be high in flow recirculation zones where residence time was large. The complex coagulation process was represented by a single variable ‘coagulant’. This model not only reduced the computational burden compared to kinetics-based model, but also produced satisfactory results when applied to patient-specific type B aortic dissections (Menichini et al. 2016, 2018). Further modification of this model was made by Armour et al. (2022) where a shear-dependent diffusive equation was introduced to avoid the deposition of coagulant on the wall with high shear rates. Taylor et al. (2016) developed a macroscopic model to simulate device-induced thrombosis and assumed thrombus deposition, and growth only occurred in low WSS regions. Their model was used to predict thrombus development in a backward facing step (BFS) geometry, and predicted results were in good agreement with the measured thrombus evolution over time using magnetic resonance imaging (Taylor et al. 2016).

However, thrombosis is a dynamic process where thrombus formation and breakdown can occur simultaneously. While most hemodynamics-based thrombosis models consider the role of low shear stress in the initiation and growth of thrombus, they often ignore the effect of thrombus breakdown induced by elevated shear stress. Taylor et al. (2016) first considered thrombus breakdown during growth, with the breakdown process being activated by a step function when shear stress on the thrombus surface exceeded a predefined threshold. This step function led to sharp boundaries in regions where local shear stress straddled the threshold (Taylor et al. 2016). An alternative switching function was adopted by Yang et al. (2021) to regulate thrombus breakdown and avoid sharp boundaries. However, the quantitative effect of thrombus breakdown on predicted thrombus growth is still unclear, especially in complex geometry under physiologically realistic flow conditions.

In this paper, a new shear stress-induced thrombus breakdown function is proposed and implemented in our previously published thrombosis model (Armour et al. 2022). The refined thrombosis model is used to predict thrombus development in a BFS under steady flow and a patient-specific AD geometry under pulsatile flow conditions. Simulations results are validated against the corresponding in vitro experimental data (Yang et al. 2021) and in vivo data acquired from an AD patient. Comparisons are also made between the refined model and the original model to quantify the effect of thrombus breakdown on the evolution of thrombus.

## Methods

### Thrombosis model

The hemodynamics-based thrombosis model first developed by Menichini and Xu (2016) was modified to incorporate shear stress-induced thrombus breakdown. This model used five transport species to simplify the thrombosis process: residence time (RT), resting platelets (RP), activated platelets (AP), coagulant (C) and bound platelets (BP). A convection–diffusion–reaction equation (Eq. 1) is used to model AP and RP:

1 $$\begin{aligned} \frac{\partial c_{i}}{\partial t}+\textbf{u}\cdot \nabla c_{i} = D_{i} \nabla ^2 c_{i}+S_{i}, i =\textrm{AP}, \textrm{RP} \end{aligned}$$

where $c_i$ is the concentration of species *i*, **u** is the flow velocity, $D_i$ is the diffusivity of species *i*, and $S_i$ is the reaction source term of species *i*, which accounts for the conversion of platelets from the resting state to the activated state caused by the exposure of RPs to both APs and thrombin. A shear-dependent diffusive equation (Eq. 2) is used to model coagulant, and this equation allows coagulant to be convection-dominant in the bulk flow and diffusion-dominant in the near wall region at low shear rates. More details of the mathematical equations can be found in Menichini and Xu (2016) and Armour et al. (2022).

2 $$\begin{aligned}{} & {} \begin{aligned} \frac{\partial C}{\partial t}=&\nabla \cdot (D_\mathrm{C\textrm{eff}} \nabla C) +k_{C}\phi _\textrm{BP}\mathrm{[AP]}\\&-k_{C_2}(1- \phi _{{{\dot{\gamma }}}})\phi _C\mathrm{[AP]} \end{aligned} \end{aligned}$$

3 $$\begin{aligned}{} & {} D_{C\textrm{eff}} = \phi _{{{\dot{\gamma }}}}D_c \end{aligned}$$

4 $$\begin{aligned}{} & {} \phi _{{{\dot{\gamma }}}} = \frac{{{\dot{\gamma }}}_t^2}{{{\dot{\gamma }}}^2+\dot{\gamma }_t^2} \end{aligned}$$

Thrombus is represented by BP, and modification is made to the original BP transport equation (Eq. 5) by introducing a breakdown term to simulate the dynamic process of thrombus growth (Eq. 6). The first term on the right-hand side of Eq. 6 allows thrombus to grow in areas with high concentration of coagulant ($\phi _c$), high residence time ($\phi _\textrm{RT}$) and high shear strain rate ($\phi _{{\dot{\gamma }}}$), where $\phi _i$ works as a switching function and is defined in Eq. 7. The growth rate is controlled by the reaction rate $K_\textrm{BP}$ and local concentration of AP. The second term on the right-hand side of Eq. 6 allows thrombus to breakdown when local shear stress on the thrombus element, $\tau _\textrm{thrombus}$, is over a fixed threshold $\tau _t$. Shear stress on a fluid element is calculated based on the viscous stress tensor defined in Eq. 8. $K_\textrm{breakdown}$ is the breakdown rate of thrombus caused by high shear stress. Parameter values have been chosen either from existing experimental measurements, such as $D_\textrm{AP}$ and $D_\textrm{RP}$ (Wootton et al. 2001), or adjusted based on sensitivity tests and several validation studies of the original thrombosis model using patient-specific computed tomography (CT) data (Menichini and Xu 2016; Armour et al. 2020, 2022; Menichini et al. 2016). All values are given in Table 1.

5 $$\begin{aligned}{} & {} \frac{\partial \textrm{BP}}{\partial t} =K_\textrm{BP}\phi _{c}\phi _\textrm{RT}\phi _{{\dot{\gamma }}}[AP] \end{aligned}$$

6 $$\begin{aligned}{} & {} \begin{aligned} \frac{\partial \textrm{BP}}{\partial t} = K_\textrm{BP}\phi _{c}\phi _\textrm{RT}\phi _{{\dot{\gamma }}}\mathrm{[AP]}\\ -K_\textrm{breakdown}\phi _\textrm{BP}\frac{\tau _\textrm{thrombus}^2}{\tau _\textrm{thrombus}^2+\tau _t^2} \end{aligned} \end{aligned}$$

7 $$\begin{aligned}{} & {} \phi _i=\frac{X_i^2}{X_i^2+X_t^2}, i = \textrm{BP}, C, \textrm{RT} \end{aligned}$$

8 $$\begin{aligned}{} & {} \tau = \sqrt{\frac{1}{6} \sum (\sigma _{ii}-\sigma _{jj})^2+ \sum \sigma _{ij}^2} \end{aligned}$$

Formed thrombus is modeled as a porous medium, and its porosity $\varepsilon$ is defined as in Eq. 9, which has a value between 0.75 (complete thrombosis) and 1 (no thrombus) (Diamond 1999). The modified Navier–Stokes equation (Eq. 10) is used which contains a negative fictitious force term $\textbf{F}$ to account for the effects of growing thrombus on blood flow. The fictitious force $\textbf{F}$ (Eq. 11) is proportional to the concentration of BP.

9 $$\begin{aligned}{} & {} \varepsilon = \max \left(1-\frac{\textrm{BP}^2}{\textrm{BP}^2+\textrm{BP}^2_t},0.75\right) \end{aligned}$$

10 $$\begin{aligned}{} & {} \begin{aligned}&\rho \frac{\partial \varepsilon \textbf{u}}{\partial t} +\nabla \cdot (\rho \varepsilon \textbf{u} \times \textbf{u})\\&= - \nabla p+\nabla \cdot (\mu (\nabla u+\nabla u^T))-\textbf{F} \end{aligned} \end{aligned}$$

11 $$\begin{aligned}{} & {} \textbf{F} = \varepsilon k_M \frac{\textrm{BP}^2}{\textrm{BP}^2+\textrm{BP}^2_t}{} \textbf{u} \end{aligned}$$

### Computational details and model validation

**Fig. 1:**
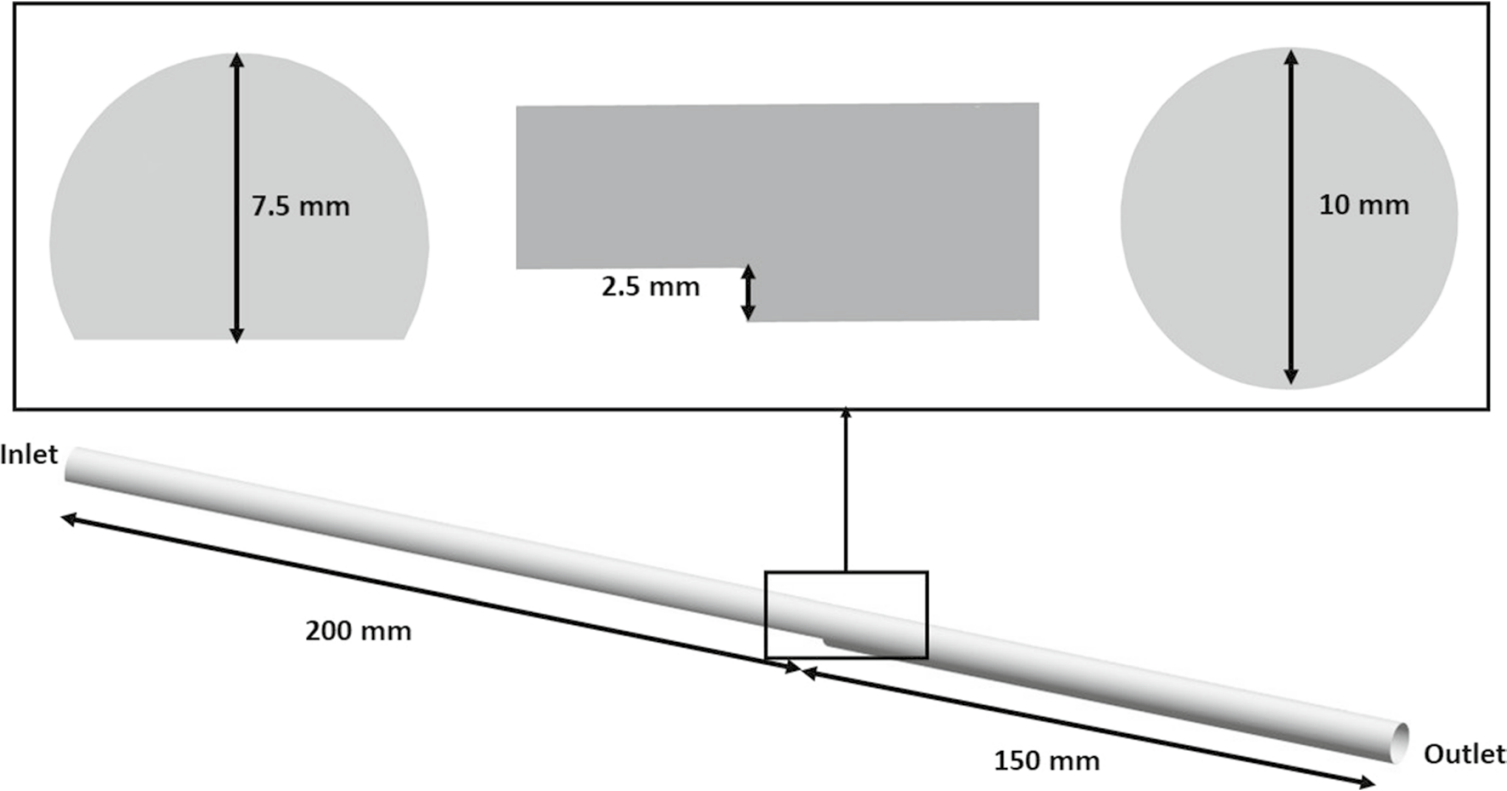
Backward facing step geometry based on dimensions provided in Yang et al. (2021)

The thrombosis model described above was implemented in Ansys CFX 19.0 (Ansys Inc) by utilizing the built-in expressions and additional variable functions. A BFS geometry, which had the same dimensions as that used by Yang et al. (2021) and Taylor et al. (2014) in their in-vitro experiments, was generated using Solidworks 2021 and then imported into ICEM 19.0 (Ansys Inc) for meshing. A mesh of 3 million elements, consisting of ten prism layers and a hexahedral core, was generated and utilized in all simulations. Details of the BFS geometry are shown in Fig. 1. To reproduce the experimental conditions in Yang et al. (2021), a flat velocity profile of a constant velocity of 0.2 m/s (Re = 490 at a dynamic viscosity of 0.0035 Pa s) was applied at the BFS model inlet, a zero-pressure boundary condition was set at the outlet, and non-slip conditions were specified at all rigid walls. Blood was modeled as a non-Newtonian fluid, and the Bird-Carreau model (Eq. 12) was used to describe its shear-thinning viscous behavior:

12 $$\begin{aligned} \mu = \mu _\infty +(\mu _0-\mu _\infty )[1+(\lambda {\dot{\gamma }})^2]^\frac{n-1}{2} \end{aligned}$$

where the high-shear viscosity $\mu _\infty$= 0.0035 Pa s, the low-shear viscosity $\mu _0$ = 0.056 Pa s, the time constant $\lambda$ = 3.313 s, and power law index n = 0.3568 (Cho and Kensey 1991). Simulations were performed with and without including the thrombus breakdown term. A timestep of 0.01 s was adopted based on the work reported by Taylor et al. (2016). All simulations were run until a stable thrombus volume (zero growth rate in thrombus volume) was reached. The simulation results were compared with the magnetic resonance imaging data of Yang et al. (2021) in terms of thrombus length, height and volume.

In addition, an anatomically faithful type B aortic dissection (TBAD) model was built based on CT scans acquired from a patient after thoracic endovascular repair of TBAD. The geometric model and computational mesh were described in a previous study (Armour et al. 2020). Boundary conditions for this model included a pulsatile flow waveform at the ascending aorta inlet and three-element Windkessel model (3-EWM) at each outlet with the relevant parameters taken from the literature (Dillon-Murphy et al. 2016). Simulations were run with a timestep of 0.005 s over 20 cardiac cycles until a stable thrombus volume was reached. Qualitative comparison of predicted thrombus volume and location was made with the follow-up CT scan of the same patient.

## Results

### Thrombus formation and growth in the BFS Model

**Fig. 2:**
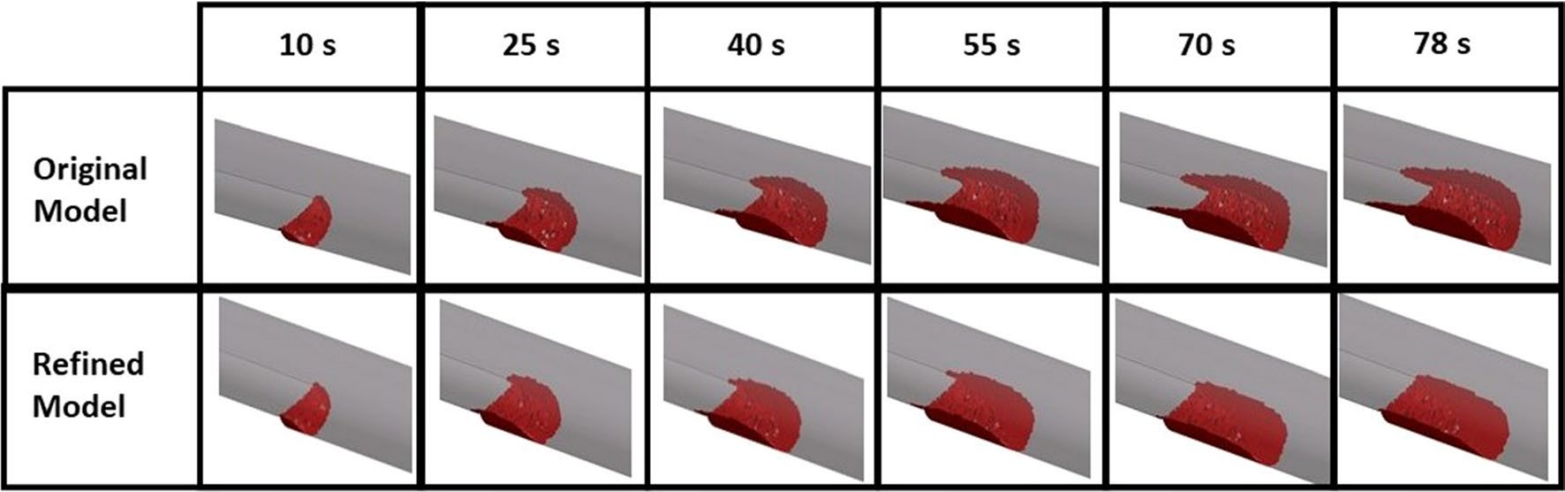
Predicted thrombus formation over time in the BFS geometry using the original (without thrombus breakdown) and refined (with thrombus breakdown) models

To validate the refined model and to quantify the effect of thrombus breakdown on predicted thrombosis, the experimental conditions reported by Yang et al. (2021) were reproduced, and their experimental results were used for comparison. Figure 2 shows the evolution of thrombus obtained with the refined and original thrombosis models. Both models predicted the initiation of thrombus formation at the step corner from where the thrombus grew in length and height. The original model predicted thrombus growth over the step, resulting in two small tails along the edges of the flat surface. The two tails extended over time and reached a length of 7.03 mm at the end of the simulation. While the refined model predicted similar thrombus formation on the step in the initial stage (25 s), the tails along the edges did not grow over time and began to disappear after 55 s.

**Fig. 3:**
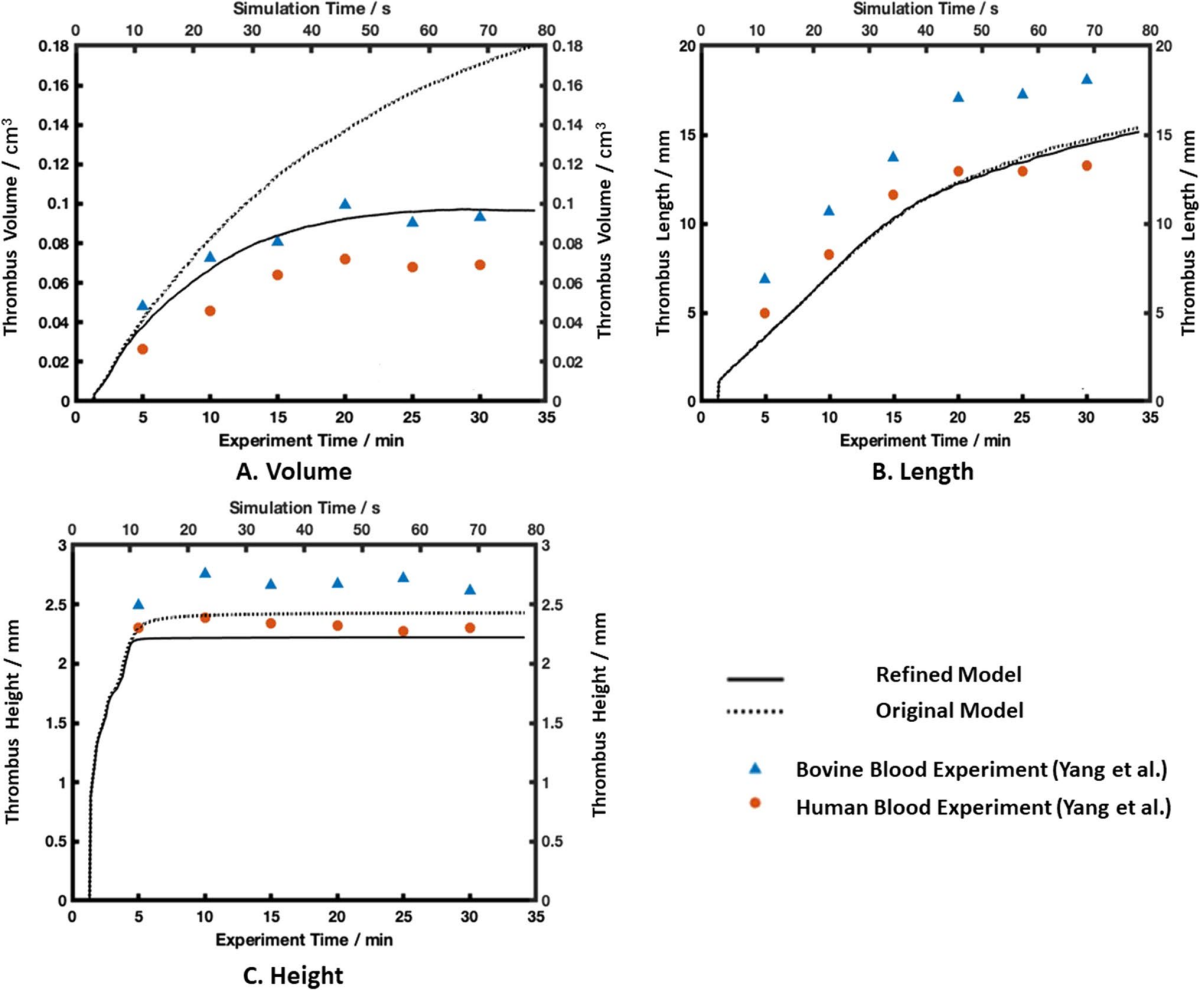
Comparisons of predicted thrombus volume (**A**), length (**B**) and height (**C**) obtained with the original (dotted line) and refined models (solid line) with the MRI experimental data provided by Yang et al. (2021) for both bovine and human blood

Quantitative comparisons of the predicted thrombus volume, height, and length were made between the two models, and with the experimental results of Yang et al. (2021). As shown in Fig. 3A, thrombus started to appear at around 3 s in the simulation time window. Without the regulation of thrombus breakdown, predicted thrombus volume increased continuously within the simulation time window. When the effect of thrombus breakdown was included, the refined model predicted a fast growth rate in the first 10 s of the simulation, after which thrombus growth slowed down and effectively stopped after 60 s.

The length and height of the predicted thrombus were measured over time and are shown in Fig. 3B, C, respectively. Both models predicted almost identical results for thrombus length, which extended almost linearly within the first 40 s of the simulation time window and at a lower rate afterward. Thrombus breakdown did not have a notable effect on the predicted length. Looking at thrombus height, both models presented a high growth rate initially when thrombus started to deposit at the step corner (Fig. 2), and this rapid growth stopped suddenly at around 10 s into the simulation when the formed thrombus reached the height of the step (2.5 mm). After this, the thrombus height hardly changed, but the maximum height predicted by the two models differed slightly, with the original model predicting a slightly larger height (2.42 mm) than the refined model (2.22 mm).

Compared to the measurements made in Yang et al.’s experiments with human and bovine blood (Yang et al. 2021), the simulation results from both models captured the same trend as experiments, except that the original model failed to predict the slowing down in thrombus growth (Fig. 3A) after the initial rapid growth phase. Quantitative comparisons revealed that both models predicted the thrombus length and height very well, but the thrombus volume obtained with the refined model was in much better agreement with the experimental measurements. Further detailed comparisons are given in Table 2, where the final thrombus volume, length and height were calculated by averaging the experimental data at 20, 25 and 30 min, and compared with the corresponding simulation results. It can be noted that the predicted thrombus length and height were in better agreement with the human blood experimental data, but the predicted thrombus volume was closer to the measurement made with bovine blood. Possible reasons for the observed discrepancies will be discussed later.

### Predicted thrombosis in TBAD

**Fig. 4:**
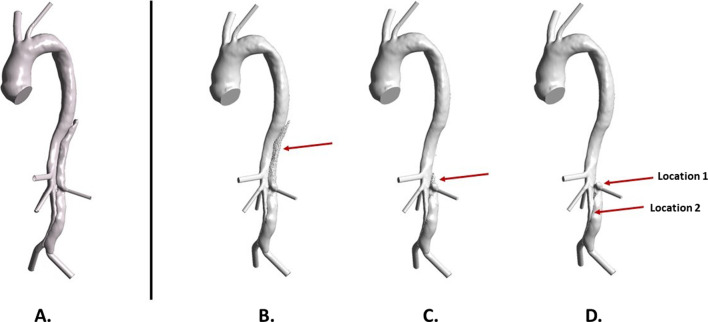
Predicted thrombosis in a patient-specific type b aortic dissection geometry using the refined model. **A** Reconstructed lumen surface based on the initial scan, and predicted change in lumen surface following false lumen thrombosis at **B** 7 cycle, **C** 13 cycle, and **D** 20 cycle

**Fig. 5:**
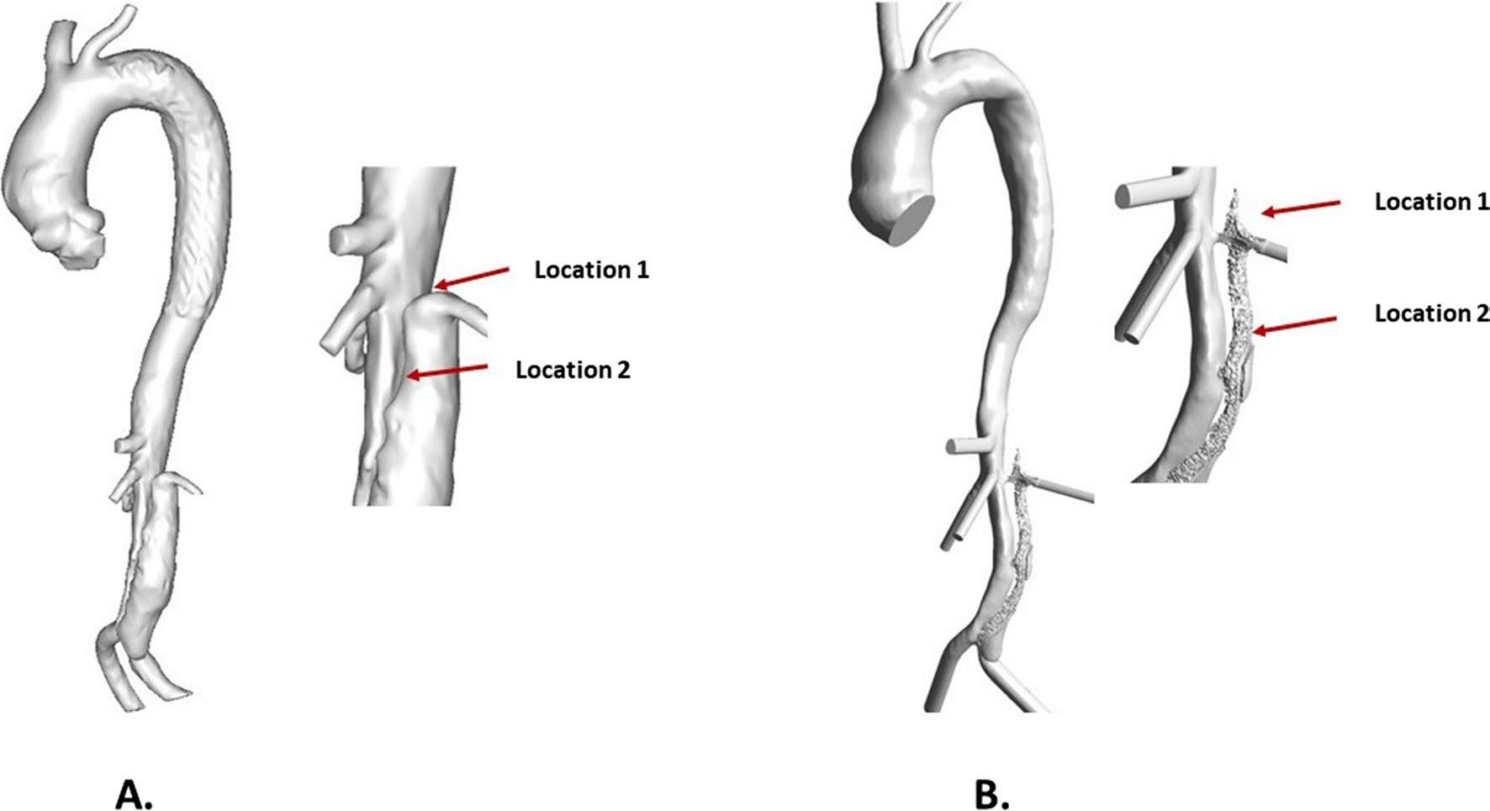
Comparison of false lumen thrombosis with in vivo measurement. **A** Reconstructed geometry at 3-year follow-up. **B** Predicted lumen surface by the original model which did not account for thrombus breakdown

Figure 4 shows changes in the FL following thrombosis predicted by the refined model. Thrombus started forming from the proximal end of the FL and gradually expanded toward the distal remaining tear. After 13 cardiac cycles in the simulation time window, most of the thoracic FL was thrombosed, and at the end of the simulation (20 cycles), the remaining FL above the right renal artery was completely thrombosed. Compared to the real FL geometry reconstructed from the 3-year follow-up CT scan (Fig. 5A), the refined model accurately captured the location and extent of thrombosis. At location 1, the FL was completely thrombosed, which was well-predicted by the refined model but not fully captured by the original model (Fig. 5B). At location 2, partial thrombosis in the FL was captured by both models, but the original model overpredicted thrombus growth.

**Fig. 6:**
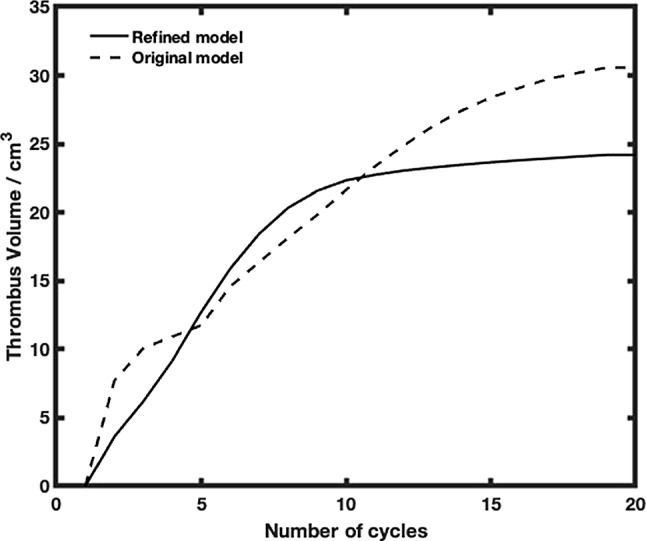
Change in thrombus volume over time obtained with the original model (without thrombus breakdown) and refined model (with thrombus breakdown)

In addition, changes in the predicted FL thrombus volume were compared between the two models. As shown in Fig. 6, a smooth growth curve was obtained with the refined model which predicted an almost linear and fast growth rate up to approximately eight cardiac cycles, and a dramatic slowdown afterward until growth stopped. Specifically, the refined model predicted little change in thrombus volume after 12 cardiac cycles. When the effect of thrombus breakdown was ignored, the original model predicted a faster growth rate during the initial phase, a sudden halt in growth for a short while, followed by a second growth phase until growth slowed down gradually, before ultimately beginning to plateau after 19 cardiac cycles. The final thrombus volume obtained with the refined model was 24.2 cm^3^, and the actual FL thrombus volume, which was obtained by subtracting the volume of the FL measured at 3-year follow-up from the initial FL volume, was approximately 22.3 cm^3^.

## Discussion

### Effects of thrombus breakdown on predicted thrombosis in BFS

Thrombosis is a dynamic process involving both thrombus formation and breakdown which can occur simultaneously. While low shear stress provides a favorable environment for thrombus formation, high shear stress acting on the surface of formed thrombus may cause thrombus breakdown (Riha et al. 1999; Dimitrov et al. 2020; Tippe and Müller-Mohnssen 1993). In this study, the thrombosis model developed by Menichini and Xu (2016) was further modified to capture this dynamic process by incorporating a new shear stress-controlled thrombus breakdown function. Both the original and refined models were applied to a BFS geometry and a patient-specific aortic dissection model for evaluation and comparison.

**Fig. 7:**
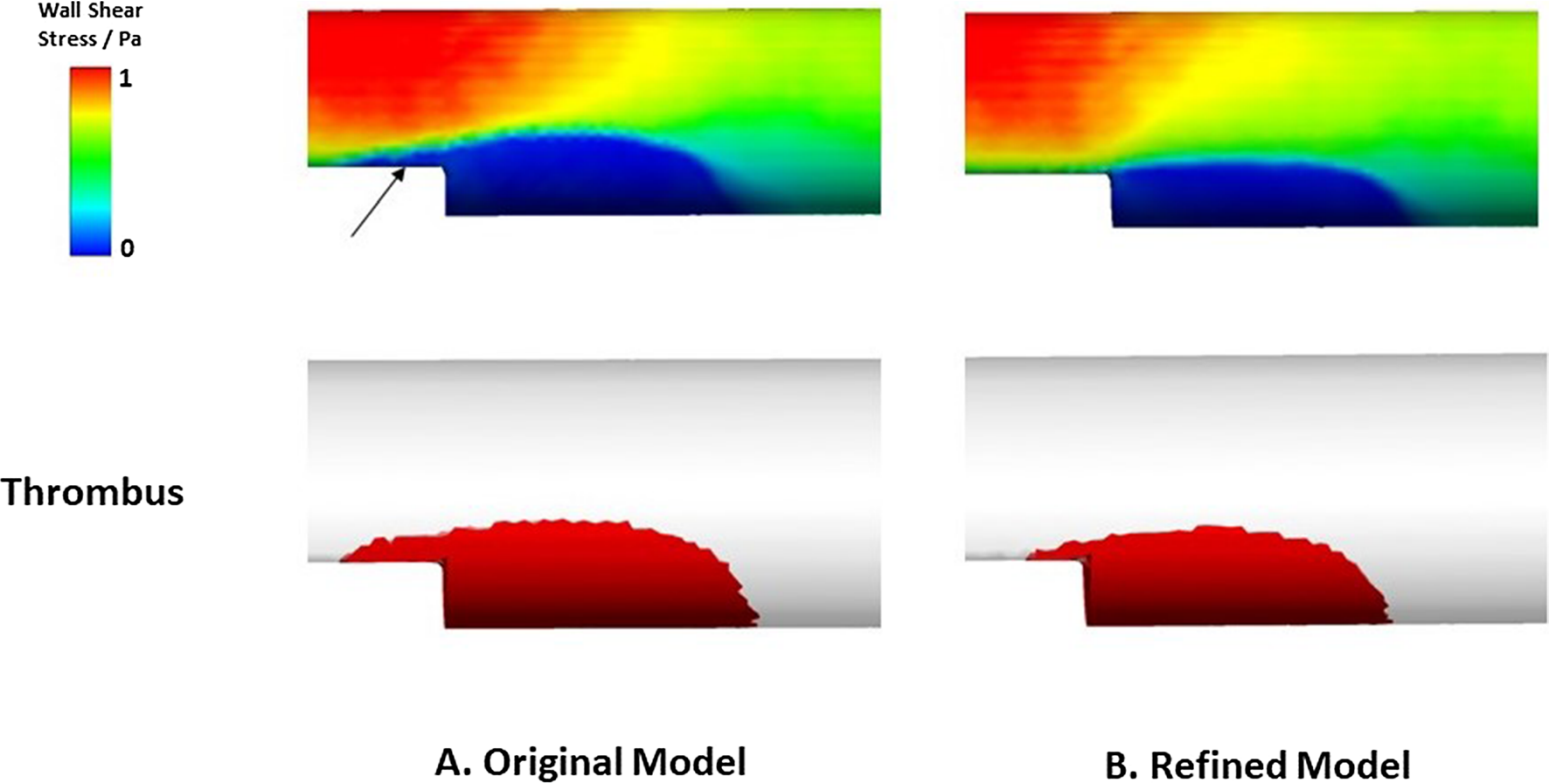
Wall shear stress distribution on the wall and the corresponding formed thrombus at 40 s by **A** the original model and **B** the refined model

In previous work (Menichini and Xu 2016; Armour et al. 2022; Jafarinia et al. 2022), a lower threshold of shear strain rate (10 s^-1^) was adopted in BFS simulations to avoid overpredictions over the step, but a value of 50 s^-1^ was used in patient-specific thrombosis modeling for both accuracy and computational efficiency. In this study, we chose the same shear rate threshold of 50 s^-1^ in all simulations. Using Menichini and Xu’s original model with this value, thrombus started to form from the step corner and grew in regions of low WSS. The thrombus growth rate was high, and no final asymptotic thrombus volume was achieved in the simulated time frame. Low WSS was observed upstream over the step (Fig. 7). As a result, thrombus formation was observed and propagated both upstream and downstream of the step in the channel. This created excessive growth that went over and beyond the step height, causing a slight bulge into the lumen. The resultant reduction in lumen area increased the local shear stress until the flow passed over the thrombus bulge.

Using the refined model, the high shear stress observed on the exposed surface of thrombus triggered the thrombus breakdown function, which helped reduce the bulge, impeded growth over and upstream of the step. As a result, a final stable thrombus volume was reached when the growth and breakdown rate reached a dynamic equilibrium. The quantitative effect of the thrombus breakdown is also reflected in the final thrombus height, reducing from 2.42 mm in the original model to 2.22 mm in the refined model.

### Comparison with experimental data in BFS

As shown in Fig. 3, the refined thrombosis model captured a smooth increase in thrombus volume, length and predicted an asymptotic shape after 50 s, which agreed with experimental measurements, but also predicted more accurate volume results compared to the original model. Quantitative comparisons summarized in Table 2 revealed that the predicted final thrombus volume agreed better with the experimental data obtained with bovine blood, while the predicted thrombus height was in better agreement with the experimental data for human blood. This inconsistency is most likely to be caused by the choice of non-Newtonian model. Although the Bird-Carreau model has been widely used to represent the shear-thinning characteristics of human blood, there are still discrepancies in shear stress values among different models (Cho and Kensey 1991; Abbasian et al. 2020; Chen and Lu 2006). At high shear strain rates (>3000 s^-1^), an asymptotic dynamic viscosity value of 0.0036 kg/ms was reached by the Bird-Carreau model, which was different from those in the experiment (Yang et al. 2021), where averaged values of 0.0035 kg/ms for bovine blood and 0.0044 kg/ms for human blood were reported. As shear stress plays an important role in the thrombosis model, this discrepancy will affect thrombus production and breakdown.

Compared to thrombus shape observed in Yang’s experiments (Yang et al. 2021), a less triangular characteristic was captured in our predicted thrombus shape, and this could be due to insufficient breakdown near the boundary. Using a different shear stress threshold for thrombus breakdown adjacent to a domain boundary should be considered in the future to help improve accuracy in predicting thrombus shape.

### Validation in patient-specific aortic dissection

Previous studies have demonstrated that the shear-driven thrombosis model is capable of predicting thrombus formation in patient-specific TBAD geometries (Menichini et al. 2016; Armour et al. 2020; Jafarinia et al. 2022). In these studies, the effect of thrombus breakdown induced by high shear stress was neglected, which could result in overpredictions of thrombus volume. By incorporating stress-induced breakdown, the refined model showed notable improvement in predicting the location and extent of thrombosis and avoided overpredicting partial thrombosis (Figs. 4 and 5). In terms of FL thrombus volume, results obtained with the refined model were within 8.5% agreement with the measurement made from follow-up CT scans, providing more confidence in future clinical applications. In addition, finding a relationship between the simulation timescale and actual thrombosis time is of interest but has not yet been addressed in previous thrombosis studies. The refined model captured a linear and smooth growth of thrombus before stabilizing (Fig. 6), and this might help derive a linear equation between thrombus volume and simulation time. As a linear relationship was also observed in the BFS experiments, it may be possible to evaluate the scaling between simulation and actual thrombosis time. However, converting the simulated thrombosis rate to the real timescale in vivo will be challenging as in vivo data cannot be acquired continuously over time.

### Limitations

Currently, this refined model has only been validated in the BFS under a steady flow condition and one patient-specific aortic dissection geometry. As the choice of inlet flow rate and pulsatile waveform has a direct influence on the magnitude of shear stress and its distribution (Armour et al. 2021), the performance of the current thrombus breakdown function should be tested for a variety of geometric and flow conditions. Validations on more complex geometries with pulsatile flows will be carried out in the future. In addition, a constant shear stress threshold value of 0.5 Pa was used to trigger thrombus breakdown in the simulations included in this study. A sensitivity analysis should be conducted in the future to evaluate the effect of this parameter on the predicted thrombus shape and size.

## Conclusion

For the first time, the current study captured a dynamic process where thrombus growth and breakdown occur simultaneously in predicting thrombosis. Our results showed that the new shear-stress regulated thrombus breakdown function was computationally efficient, and the regulation of thrombus breakdown was essential in predicting thrombus growth. In the BFS geometry, incorporating the thrombus breakdown function avoided excessive thrombus growth over the step and downstream and reduced unrealistic bulging of the thrombus surface. Moreover, it allowed the thrombus volume to stabilize more quickly. In the anatomically realistic aortic dissection geometry, the regulation of thrombus breakdown allowed the model to better predict the extent and location of thrombosis. Through qualitative and quantitative comparisons with in vitro and in vivo data, this study has demonstrated that including the effect of thrombus breakdown can improve the model performance and should be implemented in future studies.

**Table 1:** Parameter values used in thrombosis model

Parameter	Value	Reference
$X_{\textrm{RRT}_t}$	0.85	Menichini and Xu (2016) and Menichini et al. (2016)
$X_{\textrm{AP}_t}$	15	Menichini and Xu (2016) and Menichini et al. (2016)
$X_{c_t}$	10 nmol/L	Menichini and Xu (2016) and Menichini et al. (2016)
$X_{\textrm{BP}_t}$	20 nmol/L	Menichini and Xu (2016) and Menichini et al. (2016)
$\tau _t$	0.5 Pa	Empirical
$\dot{\gamma _t}$	50 s^-1^	Menichini and Xu (2016) and Menichini et al. (2016)
$$D_C$$	$$10^{-8} m^2/s $$	Savage et al. (1996), Menichini and Xu (2016) and Menichini et al. (2016)
$D_\textrm{AP}$	$1.6*10^{-13} \textrm{m}^2/\textrm{s}$	Wootton et al. (2001)
$D_\textrm{RP}$	$1.6*10^{-13} \textrm{m}^2/\textrm{s}$	Wootton et al. (2001)
$K_C$	16 nmol/L	Menichini and Xu (2016) and Menichini et al. (2016)
$K_{C_2}$	6 nmol/L	Menichini and Xu (2016) and Menichini et al. (2016)
$K_{Breakdown}$	200 s^-1^	Empirical

**Table 2:** Comparison of thrombus volume, length and height between the simulation results and experiments (Yang et al. 2021)

	Volume (mm^3^)	Length (mm)	Height (mm)
Bovine blood (Yang et al. 2021)	0.094	17.47	2.65
Human blood (Yang et al. 2021)	0.070	13.06	2.32
Refined model	0.096	15.34	2.22
